# Adherence to Patient-Reported Symptom Monitoring and Subsequent Clinical Interventions for Patients With Multiple Myeloma in Outpatient Care: Longitudinal Observational Study

**DOI:** 10.2196/46017

**Published:** 2023-08-22

**Authors:** Jens Lehmann, Kelly M de Ligt, Stefanie Tipelius, Johannes M Giesinger, Monika Sztankay, Sandra Voigt, Lonneke V van de Poll-Franse, Gerhard Rumpold, Roman Weger, Ella Willenbacher, Wolfgang Willenbacher, Bernhard Holzner

**Affiliations:** 1 University Hospital of Psychiatry II, Medical University of Innsbruck Innsbruck Austria; 2 Syndena GmbH Innsbruck Austria; 3 Department of Psychosocial Research and Epidemiology The Netherlands Cancer Institute, Antoni van Leeuwenhoek Amsterdam Netherlands; 4 Internal Medicine V: Hematology and Oncology Medical University of Innsbruck Innsbruck Austria; 5 Department of Research and Development Netherlands Comprehensive Cancer Organisation Utrecht Netherlands; 6 Department of Medical and Clinical Psychology Center of Research on Psychological and Somatic Disorders (CoRPS) Tilburg University Tilburg Netherlands; 7 Evaluation Software Development GmbH Innsbruck Austria

**Keywords:** neoplasms, patient-reported outcome measures, quality of life, ambulatory care, multiple myeloma, symptom monitoring, symptoms, monitoring, myeloma, cancer patient, therapy, application, treatment, web-based assessment, clinical care

## Abstract

**Background:**

The use of software to monitor patient-reported outcome measures (PROMs) can improve outcomes for patients with cancer receiving anticancer therapy; however, evidence from applications used in routine clinical practice is lacking.

**Objective:**

We aimed to investigate adherence to and patient perceptions of a weekly, web-based PROM symptom monitoring program in routine clinical practice for patients with Multiple Myeloma. Moreover, we aimed to capture how clinical alerts prompted by the system influenced clinical care.

**Methods:**

We conducted a single-center longitudinal observational study to evaluate patient adherence to and perceptions of the PROM monitoring software in routine practice. Patients with Multiple Myeloma remotely completed weekly treatment-specific PROMs to monitor key symptoms via a dedicated web-based platform. Alarming symptoms triggered clinical alerts in the application for the treatment team, which could initiate clinical interventions. The primary outcomes were the web-based assessment completion rate and patients’ perceptions of the monitoring program, as assessed by an evaluation questionnaire. Moreover, clinical alerts prompted by the system and consequential clinical interventions were analyzed.

**Results:**

Between July 2021 and June 2022, a total of 55 patients were approached for participation; 39 patients participated (24, 61% male, mean age 63.2, SD 9.2 years). The median assessment completion rate out of all weekly scheduled assessments was 70.3% (IQR 41.2%-89.6%). Most patients (77%) felt that the health care team was better informed about their health status due to the web-based assessments. Clinical alerts were triggered for 1758 of 14,639 (12%) reported symptoms. For 548 of 1758 (31.2%) alerts, the symptom had been registered before and no further action was required; for 348 of 1758 (19.9%) alerts, telephone consultation and self-management advice sufficed. Higher-level interventions were seldom needed in response to alerts: referral to a doctor or specialist (88/1758, 5% alerts), medication changes (22/1758, 1.3%), scheduling additional diagnostics (9/1758, 0.5%), or unplanned emergency visits (7/1758, 0.4%). Most patients (55%) reported the calls in response to alerts gave them “quite a bit” or “very much” of an added feeling of security during therapy.

**Conclusions:**

Our study shows that high adherence to regular and tailored PROM monitoring can be achieved in routine clinical care. The findings provide valuable insight into how the PROM monitoring program and the clinical alerts and resulting interventions shaped clinical practice.

**Trial Registration:**

ClinicalTrials.gov NCT05036863; https://clinicaltrials.gov/study/NCT05036863

## Introduction

In patients treated with systemic antitumor therapy, gaining optimal cancer survival benefits is balanced with managing treatment symptoms. However, clinicians tend to underestimate the symptom intensity experienced by their patients [1,2], and symptoms can go unnoticed in between clinical encounters [3,4]. Therefore, patient-reported symptom detection using patient-reported outcome measures (PROMs) gained interest over the past decades [5,6]. Clinical trials report overall survival benefits and fewer emergency room visits for cancer patients [7], hypothesizing that patient-reported symptom monitoring allows timely intervention and therefore better outcomes [8]. Moreover, the PROMs integration in clinical care can lead to improved patient-provider communication and patient satisfaction and supports patient-centered care [7,9].

The benefits of PROMs reported in clinical trials are contrasted by a lack of PROM integration in clinical routine [5,6]. Literature reports multiple challenges that prevent widespread implementation [10,11] at every step of the implementation process, including motivating clinicians to adopt PROMs and patients to complete PROMs, the technical design and compatibility with workflows, organizational issues, and adapting to issues arising during the first use by end users [10,11]. Successfully accomplishing the steps of developing, introducing, testing, integrating, and evaluating PROMs in clinical practice has been achieved in only a few centers [5,6,12]. Moreover, most symptom monitoring programs have focused on common, often solid, tumors and there is a lack of programs designed for hematological malignancies [8,13-16]; First programs are just starting to emerge [17]. Especially for hematological patients receiving outpatient care, like patients with multiple myeloma [18], who increasingly receive oral therapies, remote symptom monitoring is potentially useful. Patients have relatively regular checkups at hospitals or receive therapy there, but symptoms can go unnoticed between visits [4].

We previously developed a PROM symptom monitoring program for patients with Multiple Myeloma at the University Hospital Innsbruck, Austria [19], which is currently in use. PROMs are completed either remotely before or during patients’ visits every few weeks at the outpatient unit. We found that symptom monitoring is feasible and relevant for patients with Multiple Myeloma [19,20]. Patients’ feedback, however, suggested that more regular assessments supported by reminders and systematic screening of the results by health care professionals in-between patient visits could increase adherence and patient experience [20].

Therefore, we aimed to improve the existing symptom monitoring program at the University Hospital Innsbruck by implementing weekly, treatment-specific assessments and dedicated screening of PROM results by health care personnel who could initiate clinical interventions based on symptom reports. The study evaluated the program in terms of (1) adherence to the program, (2) patient perceptions of the program, and (3) the frequency of clinical alerts generated by the system and the nature of clinical interventions initiated in response to the alerts.

## Methods

### Setting

The study took place at the hematological outpatient unit of Internal Medicine V and the Comprehensive Cancer Center Innsbruck at the Medical University Hospital Innsbruck, Austria. PROMs are administered through the Computer-based Health Evaluation System (CHES) using a remote, web-based patient portal, in which patients complete PROMs before and during patient visits at the outpatient unit [21]. In the portal, patients can access their scores and corresponding self-management advice [20]. The PROMs assessment is part of the Austrian Myeloma Registry (AMR) [19]. The existing program was modified as follows: (1) monitoring before clinical encounters every few weeks was replaced by scheduled weekly monitoring with item lists tailored to treatment regimens, (2) reminders to prompt completion were installed, and (3) a trained onco-nurse regularly screened the PROM reports and was instructed to act upon clinical alerts triggered by the PROMs. See Multimedia Appendix 1 for detailed explanations of the monitoring program.

### Study Design

We conducted a 1-year observational longitudinal study in which patients were recruited consecutively during the study period. Patients remained on study until the predefined end date (June 30, 2022, although monitoring continued after the study) or their systemic therapy ended. The study was registered on ClinicalTrials.gov (NCT05036863) and was prepared in accordance with the STROBE (Strengthening the Reporting of Observational Studies in Epidemiology) guideline (see Multimedia Appendix 2) [22].

### Participants

We approached patients diagnosed with multiple myeloma treated at the outpatient unit. Patients were briefed and asked to sign written informed consent. Patients were eligible if they:

- had sufficient German language proficiency;
- had no overt cognitive impairments;
- were receiving active therapy;
- were able to log into a website using an individualized username and password (assessed at the initial briefing);
- reported to use the internet at least once a month.

We aimed to include all eligible patients willing to participate during the 1-year study period. Following a study board discussion on November 8, 2021, the board decided to consider the latter 2 inclusion criteria optional: patients willing to participate despite reporting rarely using the internet could participate with help from relatives. Their potential benefit from the monitoring was deemed important and outweighed the extra time and assistance the electronic patient-reported outcome (ePRO) facilitator (see below) had to invest in these patients to set them up for participation.

### Procedure and Instruments

Patients were introduced to the program by an “ePRO facilitator,” a study assistant with extensive knowledge of PROMs. Patients received patient portal login data and were instructed to complete 1 PROM assessment each week on the internet. Additionally, if patients experienced new symptoms, they could complete additional assessments at any time. If patients did not complete assessments for 7 days, automated email or text message reminders were sent. A trained onco-nurse regularly screened the completed PROM reports and acted upon clinical alerts that had been triggered based on the PROM reports. See Multimedia Appendix 1 for more information on the study procedure, PROMs used, and clinical alerts.

### Patient-Reported Outcome Measures and Clinical Alerts

In brief, the symptom monitoring consisted of symptom item lists composed of the European Organisation For Research And Treatment Of Cancer (EORTC) Item Library [23], developed following an expert-based approach. Item lists covered symptoms most relevant to patients with multiple myeloma, including a core symptom set (covering pain, fatigue, nausea, vomiting, constipation, diarrhea, polyneuropathy, emotional symptom burden, sleep disturbances, and global health) and symptoms and domains specific to patients’ treatment (eg, rash, edema, fever, or adherence to oral anticancer medication). Additionally, at baseline and every 6 weeks, comprehensive, nontreatment-specific assessments were administered, which comprised the EORTC QLQ-C30 [24] and the EORTC QLQ-MY20 [25]. For all items, Answer options range from “not at all” to “very much” (4-point Likert scale, standard EORTC item answer categories).

Clinical alerts were triggered for symptoms that exceeded 50 points on a 0 to 100 scale, with higher scores indicating higher symptom burden. This threshold corresponds to a symptom burden of “quite a bit” or “very much” (see Multimedia Appendices 1 and 3 for more information) and is in line with previous similar research [26]. For symptoms measured in the EORTC QLQ-C30, we applied the “thresholds for clinical importance” [27] to trigger alerts. In response to clinical alerts, the onco-nurse documented interventions (eg, “called patient - referred to physician”) or could register that symptoms were already documented and further intervention was not necessary.

### Program Evaluation Questionnaire

Patients received a program evaluation questionnaire after either (1) being in the symptom monitoring program for ≥6 weeks and having completed at least one remote assessment, or (2) completing 3 PROM assessments, whichever occurred first. The paper-pencil evaluation questionnaire was distributed marked with a pseudonymized ID in a prestamped envelope. The questionnaire contained questions on patients’ perceptions of the program and how it impacted their care (see Multimedia Appendix 3).

### Study Outcome Measures

See Table 1 for the study outcomes. Primary outcomes were the assessment completion rate and patients’ perceptions of the program. Secondary outcomes were the frequency and nature of clinical alerts and interventions initiated by the onco-nurse and health care team and the data completeness of the item lists.

**Table 1:** Selection of outcome measures.

Outcome		Question addressed		Assessment method
**Primary outcomes**				
	Completion rate	Can high adherence to a weekly symptom monitoring be obtained and sustained over a long time period in a routine care setting?	Function of the number of completed assessments divided by the total weeks included in the study (number of days in the study divided by 7)	
	Patient perceptions of the symptom monitoring program	How did patients experience the symptom monitoring procedure and did it affect their perception of care? (Adequacy of item lists, general experience with program, burden by questionnaires or calls, reminders)	Paper-pencil distributed evaluation questionnaire (see Multimedia Appendix 3 for details)	
**Secondary outcomes**				
	Frequency of clinical alerts for symptoms and nature of clinical interventions	How often do patients report higher symptom burden and for which symptoms? What kinds of interventions are initiated by the onco-nurse and how do they link to clinical care?	Extracted from CHES^a^: The number of symptom reports above the defined thresholds for the weekly assessments. The frequency of documented interventions by the nurse and the type of intervention (categorized).	
	Completeness of answers on item lists	Do patients complete all items on the weekly questionnaires?	Extracted from CHES: percentages of missing answers per item list and item.	

^a^CHES: Computer-based Health Evaluation System.

### Data and Statistical Analysis

Patient characteristics were extracted from the electronic health records. Data were analyzed as absolute numbers with percentages and as either means with SD if data were normally distributed or medians and IQR if not normally distributed (tested using the Shapiro-Wilk test). The results from the program evaluation questionnaire, the item lists of response completeness, and the frequency of clinical alerts and interventions by the onco-nurse are reported as absolute numbers and percentages.

The completion rate is a function of the number of completed assessments divided by the total number of weeks patients were included in the study (number of days in the study divided by 7) and reported as median and IQR. To prevent an inflated completion rate, assessments that were less than 5 days apart were considered a single assessment and counted once toward the completion rate.

### Ethics Approval

The study procedure and use of data are covered by the ethics approval for the AMR by the ethics committee of the Medical University of Innsbruck (number AN3252 266/4.2 386/5.14).

## Results

### Patient Characteristics

**Figure 1:**
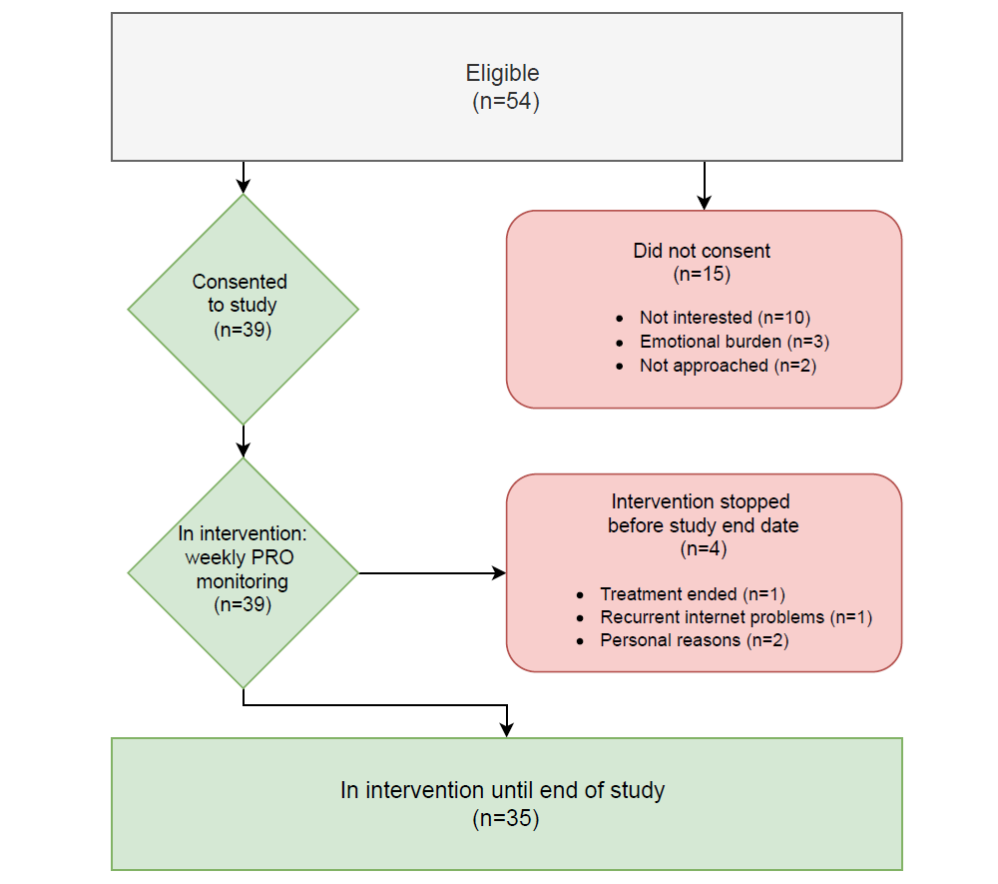
Study inclusion flowchart. PRO: patient-reported outcome.

During the study period (July 1, 2021 to June 30, 2022), 55 patients were approached; 39 (71%) patients consented to participate (Figure 1). Four patients stopped the intervention early due to recurrent internet problems at home (n=1), personal reasons (n=2), and the termination of therapy (n=1, patient excluded from study after treatment termination). Patients were on the study for a median of 316 days (IQR 232-346 days). There were 24 (62%) male patients. Patients had a mean age of 62.8 years and a median of 1534 (IQR 695-2968) days since diagnosis. Most patients (n=20, 51%) were in their first line of therapy. The most frequently used therapy was lenalidomide maintenance therapy, received by 13 (33%) patients. During the study period, 3 patients switched therapies (Table 2). As part of their past treatment (ie, before being included in the study), most patients (32/39, 82%) had previously undergone stem cell transplantation (SCT). Of those, 97% (31/32) were autologous SCT, with only one (1/32, 3%) allogenic SCT; 3 patients (3/32, 9%) had received 2 autologous SCT. No patient received a SCT during the study period.

**Table 2:** Patient characteristics at inclusion (N=39).

Characteristics			Values
**Demographics**			
	**Sex, n (%)**		
		Men	24 (61)
		Women	15 (39)
Age (years), mean (SD), range			63.2 (9.2), 44.0-88.0
	**Highest education, n (%)**		
		Compulsory or less	6 (16)
		Vocational training	16 (42)
		High school diploma	9 (24)
		University diploma	6 (16)
		Other	1 (3)
		Missing	1 (3)
**Treatment characteristics**			
	**Line of treatment, n (%)**		
		1st line	20 (51)
		2nd line	6 (51)
		3rd line	9 (23)
		4th or higher line	4 (10)
	Duration of disease in days (median, IQR)		1534 (695-2968)
	**Risk status risk (cytogenetics, or disease stage), n (%)**		
		High risk	15 (38)
		Low risk	24 (62)
	**ECOG^a^ status, n (%)**		
		0	15 (38)
		1	19 (49)
		2	5 (13)
	**Primary systemic therapy regimen during the study^b^, n (%)**		
		R mono (Lenalidomide)	13 (33)
		KRd (Carfilzomib - Lenalidomide - Dexamethasone)	7 (18)
		PdD (Pomalidomide - Dexamethason - Daratumumab)	4 (10)
		KDd (Carfilzomib - Daratumumab - Dexamethasone)	4 (10)
		Dd (Daratumumab - Dexamethasone)	4 (10)
		VRd (Bortezomib - Lenalidomide - Dexamethasone)	2 (5)
		PdE (Pomalidomide - Dexamethasone - Elotuzumab)	1 (3)
		RDd (Lenalidomide - Daratumumab - Dexamethasone)	1 (3)
		Vd (Bortezomib - Dexamethasone)	2 (5)

^a^ECOG: Eastern Cooperative Oncology Group.

^b^Multiple therapies possible if patients switched therapies. Missing data not included in the calculation of percentages.

### PROM Completion and Completion Rate

The 39 included patients completed 1047 questionnaire assessments (808 assessments with treatment specific item lists and 239 assessments with the EORTC QLQ-C30 and MY20). Of those, 13 assessments were excluded from the completion rate calculation as they were less than 5 days apart from another assessment. Patients completed an average of 26.7 (SD 13.4) unique assessments.

The median weekly assessment completion rate was 70.3% (IQR 41.2-89.6). During the study, it became apparent that weekly monitoring was too frequent for a few patients (eg, too burdensome, not appropriate for oral therapy which can have fewer side effects). To optimally support these patients, the study board decided to adjust the monitoring intervals for them. Two (5%) patients switched to a 2-weekly monitoring interval, 2 (5%) patients switched to a 3-weekly monitoring interval, and 3 (8%) patients switched to a monthly monitoring interval. However, we still considered a weekly interval for the completion rate for all patients as prespecified in the trial registration.

Over the course of the study, patients showed stable PROM assessment completion; additional analyses (see Multimedia Appendix 4) shows that the number of days between individual’s assessments did not increase significantly. In other words, patients did not tend to space out assessments further the longer they were longer in the study and mostly adhered to the 7-day assessment schedule.

Despite having the option to skip answers in the questionnaires, patients rarely did so; below 1% of the answers were missing for all item lists.

### Program Evaluation Questionnaire Results

**Figure 2:**
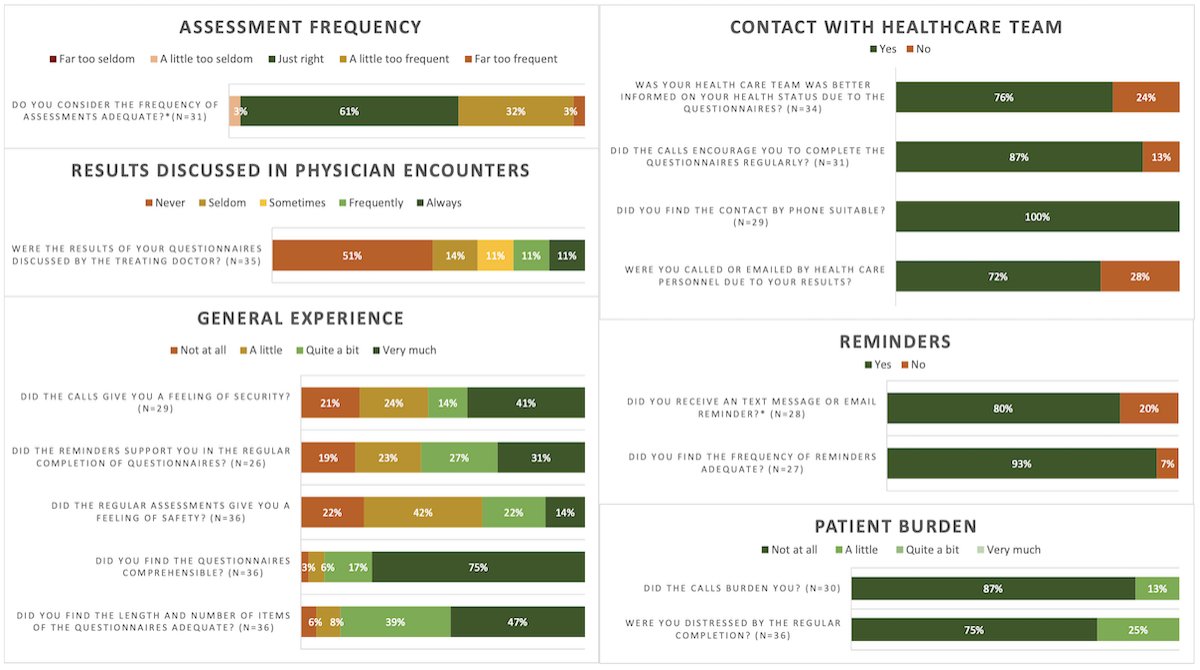
Evaluation questionnaire results (n=36).

The program evaluation questionnaire was completed by 36/39 (92%) participants. Two (5%) patients did not receive the questionnaire as they did not complete assessments in the first 6 weeks; 1 (3%) patient did not return the questionnaire. The results are summarized in Figure 2 (for complete results, see Tables S1 and S2 in Multimedia Appendix 5).

Patients were “not at all” (75%) or only “a little” (25%) burdened by regularly completing questionnaires. Most patients (78%) felt at least “a little” to “very much” safer due to the regular PROM assessments, and 55% of patients reported a feeling of security instilled by the follow-up phone calls. In total, 78% felt that the health care team was better informed on their health status due to the monitoring program. All (100%) patients felt that contact by phone was suitable. Most patients (87%) reported that the phone calls encouraged them to complete questionnaires.

### Clinical Alerts and Interventions

Table 3 shows the number of times each symptom was reported during the weekly monitoring, if the clinical alert threshold was exceeded, and if the symptom or consequential intervention were already documented. In total, 483/1041 (46.4%) PROM assessments included at least one symptom exceeding the thresholds. Clinical alerts were triggered for 1758 of 14,639 (12%) reported symptoms (multiple symptoms from a single assessments could trigger alerts).

**Table 3:** Overview of symptom reports, clinical alerts, and responses.

Symptoms (sorted by relative occurrence above threshold)	Number of symptom reports, n^a^	Reports above threshold and clinical alert sent, n (%)	Of those: response to clinical alerts		
			Symptom already known or no intervention necessary, n (%)	Intervention initiated, n (%)	No response documented by nurse, n (%)
Dyspnea	387	112 (28.9)	21 (18.8)	33 (29.5)	58 (51.8)
Pain	1013	282 (27.8)	98 (34.8)	102 (36.2)	82 (29.1)
Fatigue	1012	216 (21.3)	89 (41.2)	85 (39.4)	42 (19.4)
Back pain	438	92 (21.0)	41 (44.6)	38 (41.3)	13 (14.1)
Sleep disturbances	1014	180 (17.8)	97 (53.9)	50 (27.8)	33 (18.3)
Diarrhea	1014	164 (16.2)	62 (37.8)	50 (30.5)	52 (31.7)
Muscle cramps	589	75 (12.7)	32 (42.7)	27 (36.0)	16 (21.3)
Emotional burden	781	66 (8.5)	17 (25.8)	21 (31.8)	28 (42.4)
Polyneuropathy	781	60 (7.7)	31 (51.7)	26 (43.3)	3 (5.0)
Nausea or vomiting	1014	57 (5.6)	12 (21.1)	12 (21.1)	33 (57.9)
Edema	572	25 (4.4)	6 (24)	10 (40.0)	9 (36)
Constipation	1012	37 (3.7)	8 (21.6)	14 (37.8)	15 (40.5)
Cough	369	13 (3.5)	5 (38.5)	6 (46.2)	2 (15.4)
Weight loss	71	2 (2.8)	0 (0)	2 (100)	0 (0)
Burning or sore eyes	571	13 (2.3)	4 (30.8)	9 (69.2)	0 (0)
Blurred vision	573	15 (2.6)	7 (46.7)	5 (33.3)	3 (20.0)
Rash	762	17 (2.2)	0 (0)	6 (35.3)	11 (64.7)
Dysgeusia	324	2 (0.6)	0 (0)	2 (100.0)	0 (0)
Fever	800	3 (0.4)	1 (33.3)	2 (66.7)	0 (0)
Infusion-related reaction	31	0 (0)	N/A^b^	N/A	N/A
Other symptoms (write-in option)	808	N/A	N/A	N/A	N/A

^a^The number of symptom reports differ per domain as item lists were tailored to treatments and not every symptom was in every assessment.

^b^N/A: not applicable as it was not possible to document an intervention for this symptom category in the electronic system, or the number of reports above the threshold was 0.

The symptoms appetite loss and financial difficulties from the EORTC QLQ-C30 were not included in this analysis as they were not part of the weekly item list assessments and deemed of lower clinical relevance. The thresholds for clinical alerts were according to the Thresholds for Clinical Importance by Giesinger et al [27] for assessment using the EORTC QLQ-C30 and scoring >50 points (0-100 scale) for all other assessments or scales.

In 196 assessments, patients reported “other symptoms” in the free text item. Table S1 in Multimedia Appendix 6 shows the categorized answers; most other-symptom-reports were on additional symptoms not covered by the questionnaires, or the open answer item was used to communicate additional information to the onco-nurse.

Table 4 shows the documented responses to individual clinical alerts by the onco-nurse. For 31.2% (548/1758) of clinical alerts, the specific symptom was already documented, remained unchanged, and no further intervention was initiated based on clinical judgement by the nurse. In 19.9% (348/1758) of clinical alerts, the nurse consulted patients about their respective symptoms via phone without initiating further clinical actions. For 5% (88/1758) of clinical alerts, the nurse referred patients to the treating physician or supportive care specialists, and for 1.3% (22/1758) of them, a medication change was documented. In response to 0.4% (7/1758) of clinical alerts, an unplanned emergency visit and for 0.5% (9/1758) of additional diagnostics (eg, computerized tomography) were scheduled. Anecdotally, we report that 2 cancer recurrences were identified following diagnostics scheduled due to remote symptom reports.

**Table 4:** Type of responses to clinical alerts during the study (N=1758).

Documented response to clinical alert		Frequency, n		Percentage of total number of clinical alerts (N=1758), (%)
Symptom documented as already known, no intervention necessary		548		31.2
**Clinical interventions**				
	Called - symptom discussed, referral to self-management advice	348	19.9	
	Called - referred to specialist (doctor, psycho oncologist, etc)	88	5.0	
	Called - issued new medication	22	1.3	
	Called - results discussed with treating doctor and appointment rescheduled	12	0.7	
	Called - scheduled unplanned emergency visit	7	0.4	
	Called - issued additional diagnostics (eg, CT^a^)	9	0.5	
	Called - patient could not be reached	28	1.6	
	Other (eg, patient emailed)	53	3.0	
	No response documented^b^	643	36.6	

^a^CT: computerized tomography.

^b^In some cases, no response was documented by the onco-nurse in the system.

## Discussion

### Overview

Our study demonstrates that high completion rates can be obtained over a longer period of time for weekly web-based PROM monitoring in routine clinical care for patients with Multiple Myeloma. During the study, 39 patients completed just over 1000 assessments, with a median completion rate of 70%. Patients generally valued the weekly assessments and reported that the monitoring offered a sense of added security during therapy. Nurse-initiated interventions ranged from consultation and referral to self-management advice to issuing prescription orders or, in rare cases, emergency referrals.

### Achieving Patient Adherence to the Monitoring in Routine Clinical Care

This study was a continuation of our previous work [19,20], in which initial patient adherence to PROM monitoring was high but declined over time. We hypothesized that sending out reminders, active screening of the PROMs, and nurse-initiated clinical interventions could improve patient engagement, as reported in other studies [11,28]. We believe the implemented changes improved patient engagement and, importantly, clinical value and patients’ perceptions of the monitoring. Our completion rate is on the higher end of those reported in existing research and, often larger, clinical trials evaluating ePRO interventions: completion rates commonly vary from 41% [29] to 64.7% [16] and up to 91.5% [15]. Patients with multiple myeloma are a relatively older patient population [30]. Lower technology literacy in older people [31] may contribute to lower completion rates. Investing more resources in patient follow-up and providing alternatives to internet-based completion (eg, interactive voice response systems) may result in higher completion rates [15]. Specifically for patients with multiple myeloma, the continuous outpatient treatment at the hospital compared to transfer to follow-up care after active treatment for other types of (solid) cancers may create more relevance for long-term participation.

### Symptoms and Interventions Documented in the System

Our study complements evidence from large-scale clinical trials with diverse populations that provide evidence of the benefits of PRO monitoring systems [8,14,29,32], with important nuanced information on how such a monitoring system can function in routine clinical practice for patients with multiple myeloma. Contrary to clinical research settings, evidence of benefits from trials in routine clinical practice settings is just starting to emerge in the literature [33]. The symptoms that triggered most alerts in our study were dyspnea, general pain, fatigue, and back pain, which is generally similar to other studies using ePRO monitoring in populations with various diagnoses [15,34]. Specifically, back pain is a common symptom in patients with Multiple Myeloma that is linked to poorer overall health and functioning [35]. This finding underlines the value of using disease-specific questionnaires that are tailored to the symptoms patients are likely to experience, which likely contributed to patients’ satisfaction with the monitoring.

In our study, an onco-nurse was employed to review the assessments and contact the patient, or ask the physician to do so. Almost 80% of our patients felt that the health care team was better informed on their health status due to the questionnaires. A similar intervention in the larger “eSMART” randomized controlled trial for patients with different types of cancers during chemotherapy [32] also generated alerts from remote PROM assessments for mild to moderate symptoms that needed to be addressed in 8 hours and emergency alerts, for which a response time of 30 minutes was prescribed. Clinicians adhered to this in 95% and 85% of alerts, respectively. These are laudable response rates, especially for critical alerts, but they were likely also the result of a resource- and effort-intensive approach (eg, required dedicated site mobile devices for the alerts and shifts to allow the response time). Our monitoring program was adapted to the outpatient unit workflow and clinical practice, which did not have resources for 24-hour active monitoring. Nurse responses to patients’ symptom reports were neither timed nor continuously screened, and patients were made aware of that. Still, the monitoring resulted in many clinical interventions that arguably improved clinical care for participating patients, which might have been delayed (or not have happened at all) without the monitoring. Notably, following additional imaging that was initiated due to clinical alerts, 2 cancer recurrences were identified.

An interesting comparison can be made to other symptom monitoring programs that also rely on nurse-initiated telephone follow-up calls (not prompted by critical PROM responses on a web-based platform). For example, patients in the CAPRI (Impact of a Monitoring Device for Patients With Cancer Treated Using Oral Therapeutics) trial [29] who received symptom monitoring were also regularly called by nurses, even if they had not reported critical symptoms in PROMs. Most interventions that were initiated here were the result of these regularly scheduled follow-up calls. This approach is more resource intensive than our approach, in which patients were only called following PROM-triggered alerts. More research is needed to evaluate best practice approaches and determine the optimal balance between regularly calling patients and relying mostly on PROM-triggered clinical alerts.

Importantly, our findings show that the most frequently documented interventions were low-effort interventions, such as documenting an already-known symptom or calling the patient and referring them to the self-management advice for mild symptoms. This is similar to other studies, where the most frequent clinical responses were telephone consultations with the patient or a caregiver and subsequent referrals to self-care [15,16,29,32,34]. Arguably, this is already a clinical intervention in itself, as it can be important and reassuring for patients to have their symptoms acknowledged by the health care team, even if no immediate clinical action is required. More than half of patients (55%) reported that the calls that were made in response to alerts (and hence the subsequent interventions) gave them at least “quite a bit” to “very much” of a sense of security. Another notable finding was that for some diagnosis-specific (eg, back pain) and treatment-specific (polyneuropathy) symptoms, interventions were initiated more frequently. For example, while polyneuropathy was not a common symptom during monitoring (clinical alerts were sent only for 7.7% (60/781) of symptom reports), further clinical interventions were initiated for 43.3% (26/60) of those alerts. Patients may develop treatment-induced polyneuropathy during some types of systemic treatments, which can have debilitating effects on patients’ quality of life [36-38]. It is important that polyneuropathy is recognized as soon as possible by the treatment team to be able to initiate the necessary adjustments to the treatment and prevent long-term neurotoxicity. A remote symptom monitoring like ours may help with the timely identification of such key symptoms during outpatient therapy and, if possible, should therefore also be constructed to capture important therapy-specific side effects.

Clinically more complex interventions in coordination with the treating physician were necessary only in a few cases, meaning that most clinical alerts can be resolved by nurses who have dedicated time to screen the results. This is similar to another study in routine care where only 6.4% of calls initiated by nurses based on ePRO monitoring required an office evaluation within 72 hours of the report [34]. These findings highlight the crucial role of nurses in remote symptom monitoring programs to screen and triage patients’ symptom reports. However, our findings also showed that for some clinical alerts, the nurse failed to document a response more frequently than for other domains. Informal study debriefing interviews revealed that these lower response rates resulted from either a lack of documentation (ie, having discussed the alert with the patient but not documented it) or an uncertainty regarding how specific alerts should be handled. The latter was mostly the case for alerts for emotional burden; we have since improved our clinical workflow to incorporate a standardized clinical screening and pathway into the program. Creating clear pathways for what action should be taken in response to which alerts, which may differ by domain, can help with standardizing and improving care. In that way, nurse-initiated telephone screening can separate urgent care needs requiring immediate physician attention from less severe symptoms that may be managed with the help of self-management advice [33]. However, this ultimately requires staff resources and should be integrated into provider payment models [39].

### Patients’ Perceptions of the Monitoring Program

When completing PROMs, patients expect that their scores will prompt the health care team to take appropriate action to manage their symptoms [40]. While results were seldom directly discussed by treating physicians, the calls and interventions initiated by the nurse in response to the clinical alerts were perceived as important by patients. For more than 3 quarters of patients, the regular assessments and calls added “a little“ to a “very much” feeling of safety, and patients felt that the health care team was better informed on their health status. This compares nicely to the 77% of patients in a similar but large-scale implementation reporting that PROM monitoring made them feel in greater control of their care and the 72% of patients who felt that it improved discussions with the care team [15]. Such findings highlight the value that ePRO monitoring brings to clinical practice from the patient perspective.

### Limitations

This study has several limitations. First, the monocentric study design limits the generalizability of our findings, and the sample size does not compare to larger clinical trials. Acknowledging these limitations, we also consider the study design that was tailored to 1 specific hospital unit a partial strength of our study; it allowed us to focus on the evaluation of the monitoring program in actual clinical practice and to generate insight into the detailed clinical interventions that resulted from the PROM monitoring with high-quality longitudinal data. A second limitation is that technology literacy is an important patient-level factor for PROM completion [31]. We initially included only patients with sufficient internet access and knowledge, which might have introduced bias in the sample. However, computer literacy is changing in elderly populations, and this effect will facilitate implementations in the future. Finally, even though we investigated interventions initiated in response to patient symptom reports, we cannot draw conclusions about the effects of the monitoring on outcomes such as symptom management or survival, for which randomized controlled trials are necessary.

### Conclusions

Our study shows that high adherence to weekly, treatment-specific PROM monitoring can be achieved in routine clinical care over longer time periods. Our findings offer valuable insight into how the monitoring shaped clinical practice, how patients with multiple myeloma perceive such a program during their care, and describe first-hand experience of the clinical management and interventions that result from using such a monitoring program in routine clinical practice.

## Appendix

Multimedia Appendix 1 Further description of the monitoring program and development of item lists.

Multimedia Appendix 2 STROBE checklist.

Multimedia Appendix 3 SymOn Program evaluation questionnaire.

Multimedia Appendix 4 Scatterplot: Days from inclusion by date difference between assessments.

Multimedia Appendix 5 Evaluation questionnaire results (n=36) on reminders and contact with the healthcare professionals.

Multimedia Appendix 6 Patients’ responses in the open text item for "other symptoms" categorized (n=196).

## Abbreviations

AMR: Austrian Myeloma Registry
CAPRI: Impact of a Monitoring Device for Patients With Cancer Treated Using Oral Therapeutics
CHES: Computer-based Health Evaluation System
EORTC: European Organisation For Research And Treatment Of Cancer
ePRO: electronic patient-reported outcome
PROM: patient-reported outcome measure
SCT: stem cell transplantation
STROBE: Strengthening the Reporting of Observational Studies in Epidemiology

## Data Availability

The data sets analyzed in the study at hand are available upon reasonable request from the corresponding author. However, they partially contain sensitive medical data and will therefore only be available as processed data with sensitive and potentially identifiable data redacted.

## References

[1] Laugsand EA, Sprangers MAG, Bjordal K, Skorpen F, Kaasa S, Klepstad P (2010). Health care providers underestimate symptom intensities of cancer patients: a multicenter European study. Health Qual Life Outcomes.

[2] Chandwani KD, Zhao F, Morrow GR, Deshields TL, Minasian LM, Manola J, Fisch MJ (2017). Lack of patient-clinician concordance in cancer patients: its relation with patient variables. J Pain Symptom Manage.

[3] Atkinson TM, Li Y, Coffey CW, Sit L, Shaw M, Lavene D, Bennett AV, Fruscione M, Rogak L, Hay J, Gönen M, Schrag D, Basch E (2012). Reliability of adverse symptom event reporting by clinicians. Qual Life Res.

[4] Giesinger JM, Wintner LM, Zabernigg A, Gamper EM, Oberguggenberger AS, Sztankay MJ, Kemmler G, Holzner B (2014). Assessing quality of life on the day of chemotherapy administration underestimates patients' true symptom burden. BMC Cancer.

[5] Lohr KN, Zebrack BJ (2009). Using patient-reported outcomes in clinical practice: challenges and opportunities. Qual Life Res.

[6] Anatchkova M, Donelson SM, Skalicky AM, McHorney CA, Jagun D, Whiteley J (2018). Exploring the implementation of patient-reported outcome measures in cancer care: need for more real-world evidence results in the peer reviewed literature. J Patient Rep Outcomes.

[7] Lizán L, Pérez-Carbonell L, Comellas M (2021). Additional value of patient-reported symptom monitoring in cancer care: a systematic review of the literature. Cancers (Basel).

[8] Basch E, Deal AM, Kris MG, Scher HI, Hudis CA, Sabbatini P, Rogak L, Bennett AV, Dueck AC, Atkinson TM, Chou JF, Dulko D, Sit L, Barz A, Novotny P, Fruscione M, Sloan JA, Schrag D (2016). Symptom monitoring with patient-reported outcomes during routine cancer treatment: a randomized controlled trial. J Clin Oncol.

[9] Gibbons C, Porter I, Gonçalves-Bradley DC, Stoilov S, Ricci-Cabello I, Tsangaris E, Gangannagaripalli J, Davey A, Gibbons EJ, Kotzeva A, Evans J, van der Wees PJ, Kontopantelis E, Greenhalgh J, Bower P, Alonso J, Valderas JM (2021). Routine provision of feedback from patient-reported outcome measurements to healthcare providers and patients in clinical practice. Cochrane Database Syst Rev.

[10] Nguyen H, Butow P, Dhillon H, Sundaresan P (2021). A review of the barriers to using patient-reported outcomes (PROs) and patient-reported outcome measures (PROMs) in routine cancer care. J Med Radiat Sci.

[11] Foster A, Croot L, Brazier J, Harris J, O'Cathain A (2018). The facilitators and barriers to implementing patient reported outcome measures in organisations delivering health related services: a systematic review of reviews. J Patient Rep Outcomes.

[12] Warrington L, Absolom K, Conner M, Kellar I, Clayton B, Ayres M, Velikova G (2019). Electronic systems for patients to report and manage side effects of cancer treatment: systematic review. J Med Internet Res.

[13] Cannella L, Efficace F, Giesinger J (2018). How should we assess patient-reported outcomes in the onco-hematology clinic?. Curr Opin Support Palliat Care.

[14] Denis F, Basch E, Septans AL, Bennouna J, Urban T, Dueck AC, Letellier C (2019). Two-year survival comparing web-based symptom monitoring vs routine surveillance following treatment for lung cancer. JAMA.

[15] Basch E, Schrag D, Henson S, Jansen J, Ginos B, Stover AM, Carr P, Spears PA, Jonsson M, Deal AM, Bennett AV, Thanarajasingam G, Rogak LJ, Reeve BB, Snyder C, Bruner D, Cella D, Kottschade LA, Perlmutter J, Geoghegan C, Samuel-Ryals CA, Given B, Mazza GL, Miller R, Strasser JF, Zylla DM, Weiss A, Blinder VS, Dueck AC (2022). Effect of electronic symptom monitoring on patient-reported outcomes among patients with metastatic cancer: a randomized clinical trial. JAMA.

[16] Velikova G, Absolom K, Warrington L, Morris C, Hudson E, Carter R, Gibson A, Holmes M, Holch P, Hulme C, Brown J (2020). Phase III randomized controlled trial of eRAPID (electronic patient self-reporting of adverse-events: patient information and advice)—an eHealth intervention during chemotherapy. J Clin Oncol.

[17] Efficace F, Patriarca A, Luppi M, Potenza L, Caocci G, Tafuri A, Fazio F, Cartoni C, Petrucci MT, Carmosino I, Moia R, Casaluci GM, Boggione P, Colaci E, Giusti D, Pioli V, Sparano F, Cottone F, De Fabritiis P, Ardu NR, Niscola P, Capodanno I, Leporace AP, Pelliccia S, Lugli E, La Sala E, Rigacci L, Santopietro M, Fozza C, Siragusa S, Breccia M, Fazi P, Vignetti M (2022). Physicians' perceptions of clinical utility of a digital health tool for electronic patient-reported outcome monitoring in real-life hematology practice. Evidence from the GIMEMA-ALLIANCE platform. Front Oncol.

[18] Kumar SK, Vij R, Noga SJ, Berg D, Brent L, Dollar L, Chari A (2017). Treating multiple myeloma patients with oral therapies. Clin Lymphoma Myeloma Leuk.

[19] Sztankay M, Neppl L, Wintner LM, Loth FL, Willenbacher W, Weger R, Weyrer W, Steurer M, Rumpold G, Holzner B (2019). Complementing clinical cancer registry data with patient reported outcomes: a feasibility study on routine electronic patient-reported outcome assessment for the Austrian Myelome Registry. Eur J Cancer Care (Engl).

[20] Lehmann J, Buhl P, Giesinger JM, Wintner LM, Sztankay M, Neppl L, Willenbacher W, Weger R, Weyrer W, Rumpold G, Holzner B (2021). Using the Computer-based Health Evaluation System (CHES) to support self-management of symptoms and functional health: evaluation of hematological patient use of a web-based patient portal. J Med Internet Res.

[21] Holzner B, Giesinger JM, Pinggera J, Zugal S, Schöpf F, Oberguggenberger AS, Gamper EM, Zabernigg A, Weber B, Rumpold G (2012). The Computer-based Health Evaluation Software (CHES): a software for electronic patient-reported outcome monitoring. BMC Med Inform Decis Mak.

[22] von Elm E, Altman DG, Egger M, Pocock SJ, Gøtzsche PC, Vandenbroucke JP, STROBE Initiative (2008). The Strengthening the Reporting of Observational Studies in Epidemiology (STROBE) statement: guidelines for reporting observational studies. J Clin Epidemiol.

[23] Piccinin C, Kulis D, Bottomley A, Bjordal K, Coens C, Darlington AS, Johnson C, Velikova G, Grønvold M (2022). EORTC quality of life group item library user guidelines. European Organisation for Research and Treatment of Cancer.

[24] Aaronson NK, Ahmedzai S, Bergman B, Bullinger M, Cull A, Duez NJ, Filiberti A, Flechtner H, Fleishman SB, de Haes JCM, Kaasa S, Klee M, Osoba D, Razavi D, Rofe PB, Schraub S, Sneeuw K, Sullivan M, Takeda F (1993). The European Organization for Research and Treatment of Cancer QLQ-C30: a quality-of-life instrument for use in international clinical trials in oncology. J Natl Cancer Inst.

[25] Cocks K, Cohen D, Wisløff F, Sezer O, Lee S, Hippe E, Gimsing P, Turesson I, Hajek R, Smith A, Graham L, Phillips A, Stead M, Velikova G, Brown J, EORTC Quality of Life Group (2007). An international field study of the reliability and validity of a disease-specific questionnaire module (the QLQ-MY20) in assessing the quality of life of patients with multiple myeloma. Eur J Cancer.

[26] Friis RB, Hjollund NH, Mejdahl CT, Pappot H, Skuladottir H (2020). Electronic symptom monitoring in patients with metastatic lung cancer: a feasibility study. BMJ Open.

[27] Giesinger JM, Loth FLC, Aaronson NK, Arraras JI, Caocci G, Efficace F, Groenvold M, van Leeuwen M, Petersen MA, Ramage J, Tomaszewski KA, Young T, Holzner B, EORTC Quality of Life Group (2020). Thresholds for clinical importance were established to improve interpretation of the EORTC QLQ-C30 in clinical practice and research. J Clin Epidemiol.

[28] Schoen MW, Basch E, Hudson LL, Chung AE, Mendoza TR, Mitchell SA, St Germain D, Baumgartner P, Sit L, Rogak LJ, Shouery M, Shalley E, Reeve BB, Fawzy MR, Bhavsar NA, Cleeland C, Schrag D, Dueck AC, Abernethy AP (2018). Software for administering the National Cancer Institute's patient-reported outcomes version of the common terminology criteria for adverse events: usability study. JMIR Hum Factors.

[29] Mir O, Ferrua M, Fourcade A, Mathivon D, Duflot-Boukobza A, Dumont S, Baudin E, Delaloge S, Malka D, Albiges L, Pautier P, Robert C, Planchard D, de Botton S, Scotté F, Lemare F, Abbas M, Guillet M, Puglisi V, Di Palma M, Minvielle E (2022). Digital remote monitoring plus usual care versus usual care in patients treated with oral anticancer agents: the randomized phase 3 CAPRI trial. Nat Med.

[30] Kazandjian D (2016). Multiple myeloma epidemiology and survival: a unique malignancy. Semin Oncol.

[31] Long C, Beres LK, Wu AW, Giladi AM (2022). Patient-level barriers and facilitators to completion of patient-reported outcomes measures. Qual Life Res.

[32] Maguire R, McCann L, Kotronoulas G, Kearney N, Ream E, Armes J, Patiraki E, Furlong E, Fox P, Gaiger A, McCrone P, Berg G, Miaskowski C, Cardone A, Orr D, Flowerday A, Katsaragakis S, Darley A, Lubowitzki S, Harris J, Skene S, Miller M, Moore M, Lewis L, DeSouza N, Donnan PT (2021). Real time remote symptom monitoring during chemotherapy for cancer: European multicentre randomised controlled trial (eSMART). BMJ.

[33] Di Maio M, Basch E, Denis F, Fallowfield LJ, Ganz PA, Howell D, Kowalski C, Perrone F, Stover AM, Sundaresan P, Warrington L, Zhang L, Apostolidis K, Freeman-Daily J, Ripamonti CI, Santini D, ESMO Guidelines Committee (2022). The role of patient-reported outcome measures in the continuum of cancer clinical care: ESMO clinical practice guideline. Ann Oncol.

[34] Cherny NI, Parrinello CM, Kwiatkowsky L, Hunnicutt J, Beck T, Schaefer E, Thurow T, Kolodziej M (2022). Feasibility of large-scale implementation of an electronic patient-reported outcome remote monitoring system for patients on active treatment at a community cancer center. JCO Oncol Pract.

[35] Ludwig H, Bailey AL, Marongiu A, Khela K, Milligan G, Carlson KB, Rider A, Seesaghur A (2022). Patient-reported pain severity and health-related quality of life in patients with multiple myeloma in real world clinical practice. Cancer Rep (Hoboken).

[36] Morawska M, Grzasko N, Kostyra M, Wojciechowicz J, Hus M (2015). Therapy-related peripheral neuropathy in multiple myeloma patients. Hematol Oncol.

[37] Major A, Jakubowiak A, Derman B (2022). Longitudinal real-world neuropathy and patient-reported outcomes with bortezomib and lenalidomide in newly diagnosed multiple myeloma. Clin Lymphoma Myeloma Leuk.

[38] Selvy M, Kerckhove N, Pereira B, Barreau F, Nguyen D, Busserolles J, Giraudet F, Cabrespine A, Chaleteix C, Soubrier M, Bay JO, Lemal R, Balayssac D (2021). Prevalence of chemotherapy-induced peripheral neuropathy in multiple myeloma patients and its impact on quality of life: a single center cross-sectional study. Front Pharmacol.

[39] Basch E, Wilfong L, Schrag D (2020). Adding patient-reported outcomes to medicare's oncology value-based payment model. JAMA.

[40] Campbell R, Ju A, King MT, Rutherford C (2022). Perceived benefits and limitations of using patient-reported outcome measures in clinical practice with individual patients: a systematic review of qualitative studies. Qual Life Res.

