# Supplementary material for: Adherence to Patient-Reported Symptom Monitoring and Subsequent Clinical Interventions for Patients With Multiple Myeloma in Outpatient Care: Longitudinal Observational Study
Source: J Med Internet Res. 2023 Aug 22;25:e46017. doi: 10.2196/46017 (PMC10481208; doi:10.2196/46017)
Supplement: Multimedia Appendix 1 [file jmir_v25i1e46017_app1.docx]

## Supplementary File 1: Further description of the monitoring program and development of item lists

The full description of the monitoring programme and development prior to this study can be found in Lehmann et al. 2021 (J Med Internet Res), Sztankay et al. 2019 (Eur J Cancer Care), and Lehmann et al. 2020 (Eur Med Oncol).

### History of the quality of life monitoring programme at the department

In June 2016, routine online patient reported outcome measures (PROM) assessments were implemented to supplement outpatient care at the Department of Internal Medicine V in Innsbruck for patient with multiple myeloma (MM). This registration supplements the Austrian Myeloma Registry (AMR) [13] with PROM data. In July 2017, patients with chronic lymphocytic leukemia (CLL) were added as a second patient group.

Patients are introduced to the portal by a PROM facilitator (ie, person responsible for the assessments at the hospital), a programme assistant with a background in psychology or nursing trained in the use of PROM data and motivates both patients and HCPs to use the PRO data. Patients are asked to complete PROM symptom lists weekly. The scores are then reviewed by an onco-nurse, who is instructed to act upon conspicuous results.

### Online portal

The foundation of the symptom monitoring programme at this department lies in the Computer-based Health Evaluation System (CHES): a remote, web-based patient portal through which patients complete PROMs before and during patient visits at the outpatient unit. It enables immediate processing and graphical representation of the results to the healthcare professionals (HCP) prior to the consultation. CHES consists of 1) an interface for HCPs in which patients’ scores are presented; 2) a survey interface for PROM completion at the hospital; 3) a patient portal for online PROM completion, access to scores, and corresponding self-management advice.

The patient portal consists of the following functionalities:

- Disease-specific information on CLL and MM: diagnosis, possible symptoms, possible treatments, and links to further information and self-help groups
- Symptom monitoring through PROM assessment.
- An overview of PRO scores, display graphically in colored bar charts.
- Self-management tools and information tailored to the symptoms included in the assessment.

### Study procedure flowchart

The procedure is shown in this Figure:


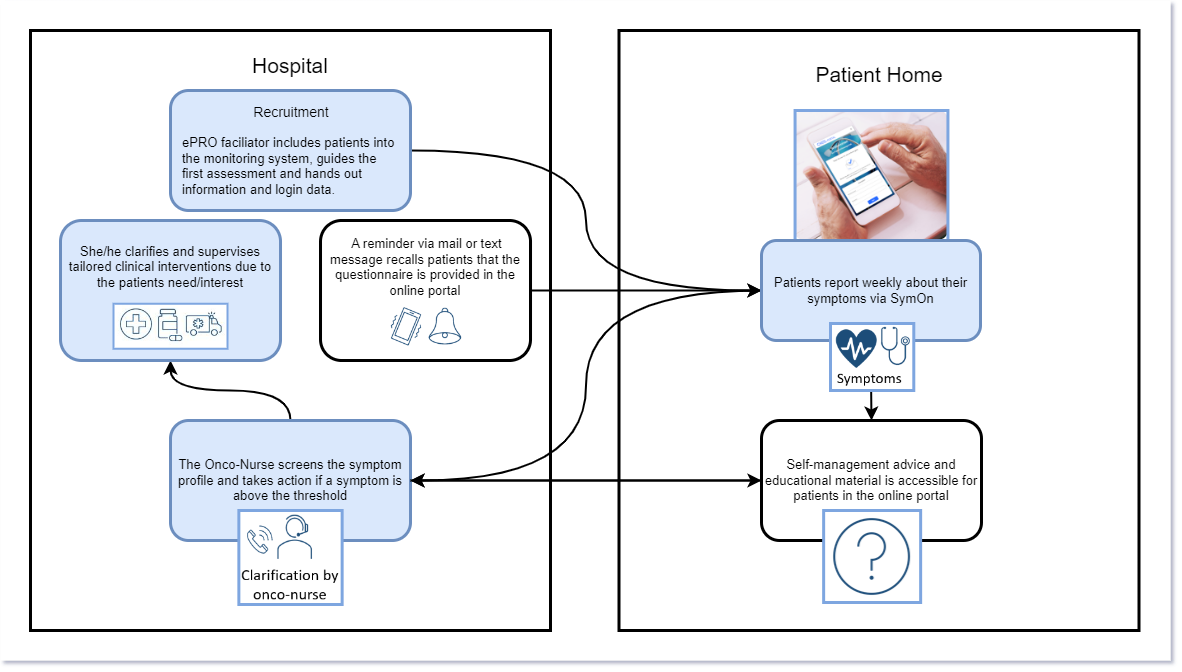


Patients were introduced to the program by a PRO facilitator, a study assistant with extensive knowledge of PROMs. Patients received login data to the patient portal and were asked to complete one PROM assessment each week via the portal. However, in case new symptoms arose, patients could complete additional assessments at any time. If patients did not complete assessments for seven days, automated email and/or text message reminders were sent. A trained onco-nurse regularly screened the PROM reports and received clinical alerts for PROMs.

### Assessments

The assessment schedule of weekly symptom assessments alternated with more comprehensive QOL assessments every 6 weeks.

The item lists for the short, weekly assessments were developed using the EORTC Item Library (https://www.eortc.be/itemlibrary/) using an expert-based approach. As a basis, we constructed a core symptom set (covering pain, fatigue, nausea, vomiting, constipation, diarrhea, polyneuropathy, emotional symptom burden, sleep disturbances, and global health) that was assessed in the same way in all patients. Then, treatment-specific symptom list were composed in an iterative process, aiming to capture the most relevant and clinically important symptoms during active therapy to maximize clinical usage. We constructed item list for the treatments most frequently used at our outpatient unit:

- VRd (Bortezomib - Lenalidomine - Dexamethason)
- KRd (Carfilzomib - Lenalidomide - Dexamethason)
- PdE (Pomalidomide - Dexamethason - Elotuzumab)
- PdD (Pomalidomide - Dexamethason - Daratumumab)
- KDd (Carfilzomib - Daratumumab - Dexamethason)
- RDd (Lenalidomide - Daratumumab - Dexamethason)
- Vd (Bortezomib - Dexamethason)
- Rd (Lenalidomide - Dexamethason)
- Dd (Daratumumab - Dexamethason)
- R mono (Lenalidomide)

Treatment-specific item lists were developed using the following process:

1. A review of the literature was conducted to gather adverse events that were self-reported in clinical trials that led to FDA approval for the respective treatment regimens described in those trials;
2. Adverse events were already covered by the basic screening questionnaire were removed;
3. We searched the EORTC Item Library for items covering adverse events/symptoms identified in the trials that could be self-reported. Items from a single scale were prioritized to ensure consistency and validity and to allow for easier scoring
4. Breadth and depth of content (as much as possible) were reviewed - if possible, selected symptom were measured using 1 easy and 1 hard item (i.e., item difficulty)
5. A consensus panel of multiple stakeholders (one hematologist, one onco-nurse, two PRO researchers, and a clinical expert from the AMR) discussed the symptoms and relevant additional issues for each treatment group;
6. Additionally, healthcare professionals on the panel had the possibility to add supposedly missing but relevant symptoms to the item lists if they were not identified in the literature search.

For the weekly symptom assessments, the following items relevant to MM were selected from the EORTC item library: back pain, pain, fatigue, sleep disturbances, muscle cramps, diarrhoea, emotional symptom burden, polyneuropathy, edema, dyspnoea, cough, weight loss, blurred vision, constipation, rash, low oral medication adherence, burning or sore eyes, nausea and vomiting, dysgeusia, fever, and infusion related reactions. We also provided an open item format where patients can enter other symptoms. Answer categories were following a 4-point Likert scale: ‘none’, ‘a little’, ‘quite a bit’, and ‘very much’. The items from the EORTC Item Library were constructed as Item Lists. As they are copyrighted by the EORTC, we cannot print them in full. However, they are available for review for anyone with access to the Item Library. The item list numbers are: IL209 to IL 221.

Additionally to the EORTC Items, we added supplementary items to assess additional symptom-specifications as follows:

- To assess adherence to oral anticancer medication, we added a question “During the last 7 days, did you take your oral anticancer medication as prescribed?” The item had different answer categories consisting of ‘did not take medication at all’, ‘took some of my medication’ and ‘took medication as prescribed’.
- To further assess fever (if a patient responded with at least “a little” on the EORTC item, we also gave an additional item where patients could enter their measured temparete in Celsius (if they had taken their temperature).
- To assess any potentially missed symptoms, at the end of every item list, a final question was given: “During the last 7 days: Did you experience any symptoms that were not covered in the questions you just answered?”. Answers were “yes” and “no”. If patients answere “yes”, they could enter their symptom in a free-text entry and then rate on a 4-point Likert scale: ‘none’, ‘a little’, ‘quite a bit’, and ‘very much’ how much the symptom bothered them during the last 7 days.

The large, comprehensive assessments included the EORTC QLQ-C30 [18] and the EORTC QLQ-MY20 [19]. These were completed at baseline and subsequently every 6 weeks. The EORTC QLQ-C30 consists of 30 items that are converted into 15 scales: global HRQoL, functioning (physical, role, emotional, cognitive, and social), and symptoms (fatigue, nausea/vomiting, pain, dyspnoea, insomnia, appetite loss, constipation, diarrhoea, and financial difficulties). Response options were on a 4-point Likert-type scale (not at all; a little; quite a bit; very much) and the recall period was the previous week; except for physical functioning (measured for ‘the current situation’). Following standard scoring procedures, raw scores are transformed linearly to scale scores ranging from 0 to 100. Higher scores indicate better global HRQoL and functioning, but worse symptom experience. The EORTC QLQ-MY20 is based on the same principles as the QLQ-C30, but covers four myeloma specific domains by 20 questions: disease symptoms, side effects of treatment, future perspective, and body image. The results from all PROMs are included in the Austrian Myeloma Registry.

### Reminders

After seven days of non-completion, the patient was sent a reminder notification (Email and/or text message, as per their preference). If the patient still did not complete an assessment in the two days following the initial reminder, a second reminder was scheduled on the third day. If three days after the second reminder the patient did not complete an assessment, the nurse tried to contact them by phone, recorded the reason for non-response, and reminded participants to complete the questionnaires in the future. Additionally, reminders could be set manually at the discretion of the onco-nurse.

### Clinical alerts and thresholds

Clinical alerts were defined for symptoms only as the monitoring focused on symptoms during therapy and the symptoms were more frequently assessed than functional scales.

For the QOL assessments (6-weekly) that used the EORTC QLQ-C30, the Thresholds for Clinical Importance [21] were used to trigger alerts. Such thresholds are not available for the item lists that were used in the weekly, symptom assessments. Therefore, the alerts for each symptom on the item lists were defined by consensus decision in an internal expert panel. The panel decided that, in line with previous research [20], clinical alerts would be send out for symptom reports that scored 50-100 points. This corresponds to responding ‘quite a bit’ or ‘very much’ on the EORTC 4-point answer category. In other words, the threshold was placed between ‘none’/‘a little’ and ‘quite a bit’/‘very much’ for those scales.

All absolute results above the threshold triggered clinical alerts, even if a patient scored above the threshold in continuous assessments and did not deteriorate or improve. However, in such cases, the nurse had the possibility to mark a symptom as “known” and document that no clinical action was necessary. If the symptom continued to be above the threshold for a longer time, the nurse was instructed to keep monitoring the symptom using occasional check-in calls via telephone. Clinicians were notified only when a symptom had become worse since the previous week.
