# Supplementary material for: Adherence to Patient-Reported Symptom Monitoring and Subsequent Clinical Interventions for Patients With Multiple Myeloma in Outpatient Care: Longitudinal Observational Study
Source: J Med Internet Res. 2023 Aug 22;25:e46017. doi: 10.2196/46017 (PMC10481208; doi:10.2196/46017)
Supplement: Multimedia Appendix 2 [file jmir_v25i1e46017_app2.docx]

Supplementary File 3: STROBE checklist

| **Section and Item** | **Item No.** | **Recommendation** | **Page** | **Section in manuscript** |
| --- | --- | --- | --- | --- |
| Title and abstract | 1 (a) | Indicate the study’s design with a commonly used term in the title or the abstract | 1 | Title page |
|  | (b) | Provide in the abstract an informative and balanced summary of what was done  and what was found | 3 | Abstract |
| ***Introduction*** | | | | |
| Background/rationale | 2 | Explain the scientific background and rationale for the investigation being reported | 2 | Introduction |
| Objectives | 3 | State specific objectives, including any prespecified hypotheses | 2 | Introduction |
| ***Methods*** | | | | |
| Study design | 4 | Present key elements of study design early in the paper | 2 | Study design |
| Setting | 5 | Describe the setting, locations, and relevant dates, including periods of recruitment,  exposure, follow-up, and data collection | 2-4 | Setting |
| Participants | 6 | Cohort study—Give the eligibility criteria, and the sources and methods of selection of participants. Describe methods of follow-up. | 3 | Participants |
| Variables | 7 | Clearly define all outcomes, exposures, predictors, potential confounders, and effect  modifiers. Give diagnostic criteria, if applicable | 4 | Study outcome measures; Table 1 |
| Data sources/  measurement | 8* | For each variable of interest, give sources of data and details of methods of assessment (measurement). Describe comparability of assessment methods if there  is more than one group | 3 | Procedure and instruments (including ‘patient reported outcome measures and clinical alerts’ and ‘program evaluation questionnaire’) |
| Bias | 9 | Describe any efforts to address potential sources of bias | 3 | Participants |
| Study size | 10 | Explain how the study size was arrived at | 3 | Participants |
| Quantitative variables | 11 | Explain how quantitative variables were handled in the analyses. If applicable,  describe which groupings were chosen and why | 4 | Data and statistical analysis |
| Statistical methods | 12 | (a) Describe all statistical methods, including those used to control for confounding  (b) Describe any methods used to examine subgroups and interactions  (c) Explain how missing data were addressed  (d) Cohort study—If applicable, explain how loss to follow-up was addressed  (e) Describe any sensitivity analyses | 4 | Data and statistical analysis; missing data were not a concern in this study (see ‘Results - PROM completion and completion rate’); sensitivity analyses were not performed |
| ***Results*** | | | | |
| Participants | 13* | (a) Report numbers of individuals at each stage of study—eg numbers potentially eligible, examined for eligibility, confirmed eligible, included in the study, completing follow-up, and analyzed  (b) Give reasons for non-participation at each stage  (c) Consider use of a flow diagram | 4-6 | Patient characteristics; Figure 1; Table 2; PROMs completion and completion rate |
| Descriptive  data | 14* | (a) Give characteristics of study participants (eg demographic, clinical, social) and information  on exposures and potential confounders  (b) Indicate number of participants with missing data for each variable of interest  (c) Cohort study—Summarise follow-up time (eg, average and total amount) | 5-6 | Patient characteristics; Figure 1; Table 2; PROMs completion and completion rate |
| Outcome data | 15* | Cohort study—Report numbers of outcome events or summary measures over time  Case-control study—Report numbers in each exposure category, or summary measures of exposure  Cross-sectional study—Report numbers of outcome events or summary measures | 6-7 | PROMs completion and completion rate; Program evaluation questionnaire result; Figure 2 |
| Main results | 16 | (a) Give unadjusted estimates and, if applicable, confounder-adjusted estimates and their  precision (eg, 95% confidence interval). Make clear which confounders were adjusted for and why they were included  (b) Report category boundaries when continuous variables were categorized  (c) If relevant, consider translating estimates of relative risk into absolute risk for a meaningful time period | 6-8 | PROMs completion and completion rate; Program evaluation questionnaire result; Figure 2; clinical alerts and interventions; Table 3 |
| Other analyses | 17 | Report other analyses done—eg analyses of subgroups and interactions, and sensitivity  analyses | N/A | N/A |
| ***Discussion*** | | | | |
| Key results | 18 | Summarise key results with reference to study objectives | 9 | Discussion |
| Limitations | 19 | Discuss limitations of the study, taking into account sources of potential bias or imprecision. Discuss both direction and magnitude of any potential bias | 10-11 | Limitations |
| Interpretation | 20 | Give a cautious overall interpretation of results considering objectives, limitations, multiplicity  of analyses, results from similar studies, and other relevant evidence | 9-11 | Discussion |
| Generalisability | 21 | Discuss the generalisability (external validity) of the study results | 9-11 | Limitations |
| ***Other information*** | | | | |
| Funding | 22 | Give the source of funding and the role of the funders for the present study and, if applicable, for the original study on which the present article is based | N/A | No funding was provided for this study. |
