# Supplementary material for: Adherence to Patient-Reported Symptom Monitoring and Subsequent Clinical Interventions for Patients With Multiple Myeloma in Outpatient Care: Longitudinal Observational Study
Source: J Med Internet Res. 2023 Aug 22;25:e46017. doi: 10.2196/46017 (PMC10481208; doi:10.2196/46017)
Supplement: Multimedia Appendix 3 [file jmir_v25i1e46017_app3.docx]

## Supplementary File 2: SymOn Program evaluation questionnaire

This is a translation of the original (German language) questionnaire. Of note, one of the questions, question 11 was originally worded “Were you called by healthcare personnel due to your results?” did initially not work well. We discovered after entering data from the first evaluation questionnaire that the patient misunderstood the question and was not contacted by phone but instead emailed. We therefore changed the question to “Were you called or emailed by healthcare personnel due to your results?” at that point during the study after discussion with the study team and the revised version was used in all subsequent interviews.

**Questionnaire for the evaluation of the patient portal 'SymOn'**

With our online patient portal ('SymOn'), we would like to accompany you as best as possible during your treatment and recognise relevant symptoms at an early stage. We are interested in how you rate our patient portal.

By answering the following short questionnaire, **you will help us to make the portal even better**. The answers you give in this questionnaire (unlike side effects and symptoms) will not be seen by medical staff. All answers in this questionnaire are collected anonymously and cannot be assigned to your person.

**Datum**:__________________

How would you rate the patient portal with regard to the following aspects?

| **1**. | Do you consider the frequency of assessments adequate? | Far too rare | A little too seldom | Just right | A little too often | Far too often |
| --- | --- | --- | --- | --- | --- | --- |

|  |  | Not at all | A little | Quite | Very |
| --- | --- | --- | --- | --- | --- |
| 2. | Were you distressed by the regular completion? | 1 | 2 | 3 | 4 |
| 3. | Did the regular assessments give you a feeling of safety? | 1 | 2 | 3 | 4 |
| 4. | Did you find the length and number of items of the questionnaires adequate? | 1 | 2 | 3 | 4 |
| 5. | Did you find the questionnaires comprehensible? | 1 | 2 | 3 | 4 |
| 6. | Did you have any technical problems when filling in the questionnaires? | Yes | No |  |  |
|  | If so, which ones? | __________________________________________________________________________ | | | |

| 7. | Did you receive an text message or email reminder?* | Yes | No |  |  |  |
| --- | --- | --- | --- | --- | --- | --- |
|  | *If so:*  Did the reminder help you to complete it regularly? | 1  Not at all | 2  A little | 3  Quite | 4  Very | (Have not received a reminder) |
| 8. | Did you find the frequency of the reminders appropriate? | Yes | No |  |  |  |
| 9. | Did you find the mode of reminders adequate? | Yes | No |  |  |  |
|  | *If no:*  What kind of type/mode of reminder would you have liked? | _____________________________________________  _____________________________________________  _____________________________________________ | | | | |
| 10. | Did you find the timing of reminders (6pm) adequate? | Yes | No |  |  |  |
|  | *If no:*  What time would have been more appropriate? | _____________________________________________  _____________________________________________  _____________________________________________ | | | | |

The following section is about contact with clinical staff:

| 11. | Were you called or emailed by healthcare personnel due to your results? | Yes | No |  |
| --- | --- | --- | --- | --- |
| 12. | Who contacted you? *(You can select more than one answer)* | Treating physician | Other personnel from the outpatient unit | Other persons  (Which one? _____________) |
| 13. | Reasons for phone call or email: *(You can select more than one answer)* | Reminder to complete questionnaires | Because of conspicuous results | Other reasons  (Which? _____________) |

| 14. | Did you find the contact by phone suitable? | | Yes | No |  |
| --- | --- | --- | --- | --- | --- |
|  | *If no:*  Why not? | ______________________________________________________________________________________________________________________________ | | | |

|  |  | | | | Not at all | | A little | | Quite | | | | Very | |  | | |
| --- | --- | --- | --- | --- | --- | --- | --- | --- | --- | --- | --- | --- | --- | --- | --- | --- | --- |
| 15. | Did the calls burden you? | | | | 1 | | 2 | | 3 | | | | 4 | | (not applicable) | | |
| 16. | Did the calls give you a feeling of security? | | | | 1 | | 2 | | 3 | | | | 4 | | (not applicable) | | |
| 17. | Did the reminders support you in the regular completion of questionnaires? | | | | | | | | | Yes | | No | | | | |  |
|  | *If no:*  Why not? | | _______________________________________________________  _______________________________________________________ | | | | | | | | | | | | | | |
| 18. | Did you have the impression that your healthcare team was better informed on your health status due to the questionnaires? | | | | | | | | | Yes | | No | | | | |  |
|  | *If no:*  Why not? | | _______________________________________________________  _______________________________________________________ | | | | | | | | | | | | | | |
| 19. | | Were the results of your questionnaires discussed by the treating doctor? | | | | Never | | Rare | | | Sometimes | | | Often | | Always | |
|  | | *If no:*  Why not? | | _______________________________________________________  _______________________________________________________ | | | | | | | | | | | | | |

Do you have any further criticism, comments or suggestions for improvement regarding the patient portal? If so, you can enter them here:

________________________________________________________________________

________________________________________________________________________

________________________________________________________________________
