# Supplementary material for: Adherence to Patient-Reported Symptom Monitoring and Subsequent Clinical Interventions for Patients With Multiple Myeloma in Outpatient Care: Longitudinal Observational Study
Source: J Med Internet Res. 2023 Aug 22;25:e46017. doi: 10.2196/46017 (PMC10481208; doi:10.2196/46017)
Supplement: Multimedia Appendix 4 [file jmir_v25i1e46017_app4.docx]

Supplementary File 4: Scatterplot: Days from inclusion by date difference between assessments


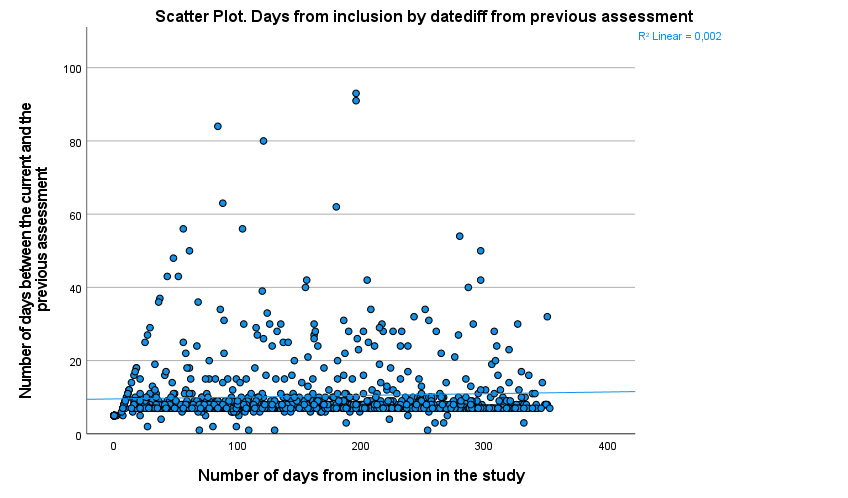


Note. The plot shows the number of days from the last assessments on the y-axis by the number of days from inclusion in the study.
