# Supplementary material for: Adherence to Patient-Reported Symptom Monitoring and Subsequent Clinical Interventions for Patients With Multiple Myeloma in Outpatient Care: Longitudinal Observational Study
Source: J Med Internet Res. 2023 Aug 22;25:e46017. doi: 10.2196/46017 (PMC10481208; doi:10.2196/46017)
Supplement: Multimedia Appendix 5 [file jmir_v25i1e46017_app5.docx]

### Multimedia Appendix 5

### Table S1. Evaluation questionnaire results (n=36) on reminders and contact with the healthcare professionals.

| **Question** | | **Yes** | | **No** | | **Missing** |
| --- | --- | --- | --- | --- | --- | --- |
|  |  | **n** | **%** | **n** | **%** | **n** |
| ***Reminder texts and emails*** | | | | | | |
| Did you receive an text message or email reminder?* | | 28 | 80.0 | 7 | 20.0 | 1 |
| Did you find the timing of reminders (6pm) adequate? | | 27 | 96.4 | 1 | 3.6 | 8 |
| Did you find the frequency of reminders adequate? | | 25 | 92.6 | 2 | 7.4 | 9 |
| Did you find the mode of reminders adequate? | | 27 | 100 | 0 | 0.0 | 9 |
| ***Contact by phone or email*** | | | | | | |
| Were you called or emailed by healthcare personnel due to your results? | | 26 | 72.2 | 10 | 27.8 | 0 |
| Who contacted you: | |  | | | | |
|  | treating physician | 7 | 21.2 | 26 | 78.8 | 3 |
|  | other personnel from the outpatient unit | 22 | 64.7 | 12 | 35.3 | 2 |
| Reasons for phone call or email: | |  | | | | |
|  | reminder to complete questionnaires | 8 | 24.2 | 25 | 75.8 | 3 |
|  | because of conspicuous results | 21 | 65.6 | 11 | 34.4 | 4 |
| Did you find the contact by phone suitable? | | 29 | 100 | 0 | 0 | 7 |
| Did the calls encourage you to complete the questionnaires regularly? | | 27 | 87.1 | 4 | 12.9 | 5 |
| Did you have the impression that your healthcare team was better informed on your health status due to the questionnaires? | | 26 | 76.5 | 8 | 23.5 | 2 |
| **Legend:**  *Out of the 7 patients that reported to not have received a reminder, 5 had received reminders (as documented by the system); later informal interviews revealed that those patients did not understand that the question regarding ‘reminders’ referred to the emails/text messages. | | | | | | |

### Table S2. Questionnaire evaluation results (n=36) on patient experience in terms of benefits and burden.

| **Question** | **A little too seldom** | | **Just right** | | **A little too frequent** | | **Far too frequent** | | **Missing** | |
| --- | --- | --- | --- | --- | --- | --- | --- | --- | --- | --- |
|  | **n** | **%** | **n** | **%** | **n** | **%** | **n** | **%** | **n** | |
| Do you consider the frequency of assessments adequate?* | 1 | 3.2 | 19 | 61.2 | 10 | 32.3 | 1 | 3.2 | 5 | |
|  | **Not at all** | | **A little** | | **Quite a bit** | | **Very much** | | **Missing** | |
|  | **n** | **%** | **n** | **%** | **n** | **%** | **n** | **%** | **n** | |
| Were you distressed by the regular completion? | 27 | 75.0 | 9 | 25.0 | 0 | 0.0 | 0 | 0.0 | 0 | |
| Did you find the length and number of items of the questionnaires adequate? | 2 | 5.6 | 3 | 8.3 | 14 | 38.9 | 17 | 47.2 | 0 | |
| Did you find the questionnaires comprehensible? | 1 | 2.8 | 2 | 5.6 | 6 | 16.7 | 27 | 75.0 | 0 | |
| Did the regular assessments give you a feeling of safety? | 8 | 22.2 | 15 | 41.7 | 8 | 22.2 | 5 | 13.9 | 0 | |
| Did the calls burden you? | 26 | 86.7 | 4 | 13.3 | 0 | 0.0 | 0 | 0.0 | 5 | |
| Did the reminders support you in the regular completion of questionnaires? | 5 | 19.2 | 6 | 23.1 | 7 | 26.9 | 8 | 30.7 | 10 | |
| Did the calls give you a feeling of security? | 6 | 20.7 | 7 | 24.1 | 4 | 13.8 | 12 | 41.4 | 7 | |
|  | **Never** | | **Seldom** | | **Sometimes** | | **Frequently** | | **Always** | |
|  | **n** | **%** | **n** | **%** | **n** | **%** | **n** | **%** | **n** | **%** |
| Were the results of your questionnaires discussed by the treating doctor? | 18 | 51.4 | 5 | 14.3 | 4 | 11.4 | 4 | 11.4 | 4 | 11.4 |
| **Legend:**  * This question had a higher rate of missing answers (5/36, 14%); this item was the first item on the questionnaire and formatted differently than the other items and patients might have overlooked it. | | | | | | | | | | |
