# Supplementary material for: Adherence to Patient-Reported Symptom Monitoring and Subsequent Clinical Interventions for Patients With Multiple Myeloma in Outpatient Care: Longitudinal Observational Study
Source: J Med Internet Res. 2023 Aug 22;25:e46017. doi: 10.2196/46017 (PMC10481208; doi:10.2196/46017)
Supplement: Multimedia Appendix 6 [file jmir_v25i1e46017_app6.docx]

## Multimedia Appendix 6

## **Table S1.** Patients’ responses in the open text item for “other symptoms” categorized (n=196).

| **Category** | **Frequency** | **Percentage** |
| --- | --- | --- |
| Reports of symptoms not covered by the treatment-specific questionnaires (eg, tooth pain) | 109 | 55.6% |
| Further explanation of the symptom trajectory of symptoms reported in the symptom questionnaire (eg, “this symptom improved in the last days”) | 62 | 31.6% |
| Reports on treatment outside of the hospital that had already been imitated (e.g., by general practitioner) or on medication that was taken | 21 | 10.7% |
| Miscellaneous communication with the onco-nurse | 4 | 2.0 |
